# Characteristics of Tuberculous Meningitis in HIV-Positive Patients from Northeast Romania

**DOI:** 10.3390/clinpract13060131

**Published:** 2023-11-21

**Authors:** Isabela Ioana Loghin, Andrei Vâță, Egidia Gabriela Miftode, Mihaela Cobaschi, Șerban Alin Rusu, George Silvaș, Otilia Elena Frăsinariu, Carmen Mihaela Dorobăț

**Affiliations:** 1 Department of Infectious Diseases, “Grigore T. Popa” University of Medicine and Pharmacy, 700115 Iasi, Romania; isabelabegezsan@yahoo.com (I.I.L.); andrei.vata@umfiasi.ro (A.V.); emiftode@yahoo.co.uk (E.G.M.); 2 Department of Infectious Diseases, “St. Parascheva” Clinical Hospital of Infectious Diseases, 700116 Iasi, Romania; rususerbanalin@yahoo.com (Ș.A.R.); silvas.george@gmail.com (G.S.); carmendorobat@yahoo.com (C.M.D.); 3 Faculty of Medicine/Clinical II Department, “Carol Davila” University of Medicine and Pharmacy, 050474 Bucharest, Romania; 4 National Institute of Infectious Diseases “Prof. Dr. Matei Balș”, 021105 Bucharest, Romania; 5 Emergency Clinical Hospital for Children “St. Maria”, 700309 Iasi, Romania

**Keywords:** HIV infection, tuberculous meningitis, immunosuppression, ART, Thwaites score

## Abstract

Background and objectives: One of the most severe forms of extrapulmonary tuberculosis (EPTB) is tuberculous meningitis (TBM), which is linked to significant morbidity and high mortality. It is well recognized that human immunodeficiency virus (HIV)-positive people are more likely to develop EPTB, including TBM, especially if they have severe immunodeficiencies. We aim to highlight the profile and the characteristics of TBM in HIV-infected patients. Material and methods: We conducted a retrospective clinical study based on hospital medical records of patients diagnosed with HIV/AIDS (acquired immunodeficiency syndrome) and TBM in Northeast Romania, hospitalized at “St. Parascheva” Clinical Hospital of Infectious Diseases of Iasi from 1 January 2010 to 1 December 2022. Results: From a total number of 1692 patients on record in our center, 195 had a HIV–tuberculosis (TB) coinfection, and 19 cases were HIV–TBM coinfected. Six cases were newly HIV-diagnosed late presenters, and thirteen patients’ names were already found in the center’s records with deficient immunological viral status (median CD4 lymphocyte level 47/mm^3^). The average age in the study group was 27 years old. The clinical manifestations and cerebrospinal fluid (CSF) variables were typical in most cases, despite the severe immunodepression of the patients. The Thwaites scoring system correctly identified 89.5% of the patients. The median admission period was 18 days; the lethality rate was 31.6%, despite access to ART and anti-TB drugs, and was associated with a more severe immunosuppression. No rifampicin resistance was detected. Conclusions: TBM appeared in a minority of our HIV cohort and affected severely immunodepressed patients; the clinical and CSF variables had a typical aspect in most cases, and the Thwaites scoring system performed well for this type of patient. The lethality rate was high and was correlated with a more severe immunodepression.

## 1. Introduction

The human immunodeficiency virus (HIV) attacks the immune system, which can lead to AIDS (acquired immunodeficiency syndrome), a condition that increases susceptibility to other infections. HIV/AIDS is a global public health issue, with an estimated 38 million people living with HIV/AIDS worldwide in 2019 [1]. Since the virus was first identified in the early 1980s, HIV/AIDS has become one of the deadliest pandemics in human history, with over 36 million people dying from AIDS-related illnesses [2]. Globally, women represent about half of all people living with HIV/AIDS; in sub-Saharan Africa, they are reported to constitute 60% of the total [3]. In 2021, in Europe, the second-most prevalent reported method of HIV transmission was heterosexual contact, accounting for 29% of HIV diagnoses. In five European countries (Estonia, France, Latvia, Norway, and Romania), heterosexual transmission was the most commonly reported known form of transmission [3]. The majority of new HIV diagnoses in Europe are among MSM (men who have sex with men), who accounted for 42% of new diagnoses in 2019. Other key populations with higher rates of HIV/AIDS in Europe include PWIDs (people who inject drugs), migrants, and sex workers. According to the World Health Organization (WHO), there were approximately 2.2 million people living with HIV/AIDS in Europe in 2019, with an estimated 136,000 new diagnoses [4]. 

Romania has a relatively low prevalence of HIV/AIDS compared to some other countries in Europe, but the epidemic has been growing in recent years. In Romania, according to “Matei Bals” National Institute for Infectious Diseases, between 1985 and 2022 there were a total of 26,554 HIV-infected patients, of whom 17,536 were periodically evaluated in the Regional HIV/AIDS Centers in Romania [5]. In 2022, the most frequent transmission route was sexual (heterosexual 59.3%, MSM 31%) followed by intravenous drug users (7.6%). HIV/AIDS was more frequent in men (81%) compared to women (19%) [5].

According to the ECDC (European Centre for Disease Control), in Romania, in 2020 there were 7698 cases of TB cases reported, with an incidence rate of 39.8 per 100,000 people. In the last 10 years, in Romania, 13.6% of all TB infections were EPTB, according to the ECDC Surveillance Atlas of Infectious Diseases [6]. One of the most severe forms of EPTB is TBM, which is linked to significant morbidity and high mortality. TBM represents 1–5% of the total TB cases worldwide [3,7]. In our region, TBM is not an exceptional diagnosis; Luca et al. reported 76 cases in a 4-year period (2008–2011) [8]. It is well recognized that HIV-positive people are more likely to develop EPTB, including TBM, especially if they have severe immunodeficiency. Additionally, it has been demonstrated that TBM patients among HIV-positive patients died at higher rates than HIV-negative people in the time before receiving combination antiretroviral therapy [7,9].

The onset of TBM may last from one day to six months, and, as a result, the condition may manifest as either acute or chronic meningitis. Fatigue, malaise, anorexia, vomiting, fever, and headache are among the symptoms of TBM’s nonspecific prodrome; symptom variation throughout this time is frequent. Occasionally, TBM may manifest as gradual dementia, with social retreat and personality abnormalities. Active pulmonary TB coexists with TBM in 30% to 60% of cases. Acute presentations may be difficult to distinguish from bacterial meningitis [10].

TBM diagnosis is based on specific symptoms and microbiological exams of CSF. Certain clinical criteria, such as symptoms lasting more than six days, and mild CSF pleocytosis raise the likelihood of TBM. CSF analysis shows a lymphocytic pleocytosis that frequently coexists with low glucose and increased protein levels [11].

TBM treatment, especially in HIV-infected persons, is complicated, even if the use of ART may improve clinical outcomes. In addition to the known drug–drug interactions, starting ART and anti-tuberculous therapy at the same time results in overlapping drug toxicities [12,13] and the appearance of a paradoxical reaction, known as immune reconstitution inflammatory syndrome (IRIS), in which the patient displays a temporary exacerbation of the signs and symptoms of TB infection when ART use begins, is a possible additional problem [14,15].

Since patients with advanced HIV infection have a particularly poor prognosis after TBM, there were urgent calls for public health interventions to improve patient management in terms of timely and accurate TB diagnoses, optimal TB treatment, and access to personalized ART [16]. 

We aimed to describe the clinical, laboratory, and therapeutic characteristics and the evolution of TBM in our HIV-infected patients over a 13-year period (between 2010 and 2022). We hope that our results can help clinicians improve their early TBM diagnoses in this type of patient and the overall prognosis for meningeal TB–HIV coinfection.

## 2. Materials and Methods

This was a retrospective clinical study based on the medical records of patients diagnosed with TBM and HIV/AIDS who were hospitalized at “St. Parascheva” Hospital of Infectious Diseases Iasi, Romania, between 1 January 2010 and 1 December 2022. Our hospital coordinates the management for HIV-infected patients in six counties of Northeast Romania, and is the regional referral center for this type of pathology. 

### 2.1. Database Description

The information gathered included demographic factors (age, sex), clinical traits, blood tests, associated opportunistic infections, patient staging according to the Centers for Disease Control and Prevention (CDC) Atlanta recommendations [17], antiretroviral treatment, and prognosis following therapy.

The diagnosis of HIV infection was established after two ELISA tests (Genscreen™ HIV-1 Ag confirmatory assay from Bio-Rad, Hercules, CA, USA) and a confirmatory Western blot test. The patients were further evaluated, and the patient’s CD4+ T cell count and HIV plasmatic viral load (RT-PCR HIV 1 Cepheid’s GeneX-pert^®^, Sunnyvale, CA, USA) were measured. 

The diagnosis of TBM was based on clinical and epidemiological data and the analysis of CSF variables (quantitative, qualitative examination, and biochemistry examination of glucose, proteins, and chlorine). *Mycobacterium tuberculosis* infection was confirmed with Xpert MTB/RIF assay PCR *Mycobacterium tuberculosis* DNA (the test simultaneously detects *Mycobacterium tuberculosis* complex and resistance to rifampin) and/or CSF cultures. 

The performance of “Thwaites’ system” [18], which takes into consideration the age of the patients (≥36 years, +2 points), white blood cell count (≥15,000/mm^3^, +4 points), history of illness (≥6 days, −5 points), the CSF white cell count (≥750/mm^3^, +3 points), and the percent of neutrophils from the CSF (≥90, +4 points), was assessed in our patients, in order to differentiate TBM from other bacterial meningitis. A total of at most 4 points was considered suggestive of TBM.

### 2.2. Statistical Analysis

The descriptive data are presented as absolute values, percentages, and means. Differences between groups were tested for statistical significance using unpaired Student’s *t*-test and χ^2^ tests. The Pearson test was used to correlate demographic factors and clinical data, and the outcome *p* < 0.05 was considered to indicate a statistically significant difference. Statistical Software for Excel (XLSTAT) version 2019 was used to conduct statistical analysis.

## 3. Results

Our HIV/AIDS Regional Center has on record a total number of 1692 HIV patients from the county regions of Iasi, Bacau, Botosani, Suceava, Neamt, and Vaslui. The counties listed above are 6 out of the total of 41 counties in Romania, in the North East region, with a cumulative population of more than 3.6 million people, taking into consideration that the population of Romania is over 19 million people. Of these HIV-infected people in our center, 195 had pulmonary or ganglionar TB, and 19 cases had TBM during the study timeframe (Table 1).

Between 2010 and 2022, “St. Parascheva” Infectious Diseases Hospital managed a total number of 697 TB-infected HIV-negative patients, of whom 93 had been diagnosed with TBM. Out of the 195 HIV–TB coinfected cases, 19 had TBM (Table 2).

Although the number of TB–HIV coinfected patients and non-HIV TBM cases significantly decreased in the last half of the studied period (2010–2015 vs. 2016–2022, an average 23 vs. 8 cases/year, *p* = 0.0027 and 12 vs. 3 cases/year, respectively; *p* = 0.0006), the number of TBM cases among HIV patients remained stable (6 cases between 2010 and 2015 vs. 7 cases between 2016 and 2022).

From the 19 TBM HIV-positive cases (10 women and 9 men) included, 13 patients were already reported to have HIV infection in the center’s records. The remaining six patients were diagnosed simultaneously with HIV and TBM. From the total number of cases, two were diagnosed in 2010, one in 2011, two in 2012, two in 2013, one in 2014, one in 2015, four in 2016, one in 2017, one in 2018, one in 2019, one in 2020, and two in 2022 (Table 2).

The median age in the TBM study group was 27 years old. The majority of TBM cases were young adults aged between 21 and 30 years old. There were significant differences in the age group distribution of TBM cases compared with the TB–HIV cases (where most belonged to the 41–50 years group) and to the HIV non-TB patients (where most belonged to the 31–40 years group) (Table 3). The median age of the non-TB HIV group was significantly higher than that of the TBM patients (36 vs. 27 years old, *p* = 0.0007). Significant differences were also noticed in the proportion of patients with a CD4 T cell count < 200/mm^3^ between the TBM and TB–HIV patients (89.5% and 91.8%) vs. the non-TB HIV patients (26%). The median number of CD4 T cells in the TBM group was 47/mm^3^.

The geographical distribution of our patients showed that almost one-third of the patients were from the county region of Suceava (seven cases, 37%), followed by Iasi (four cases, 21%), Botosani (four cases, 21%), Bacau (two cases, 11%), Neamt (one case, 5%), and Vaslui (one case, 5%). Seven patients (37%) lived in urban areas in Northeast Romania, and the remaining twelve cases (63%) were from rural areas. 

The route of HIV transmission was reported in 18 cases, including 17 cases of sexual transmission (MSM and heterosexual contact, 89.47%) and 1 case of intravenous drug usage.

Most patients (12/19) had a progressive onset of symptoms; the duration exceeded 7 days in 9/19. The median onset period was 9 days. 

**Figure 1:**
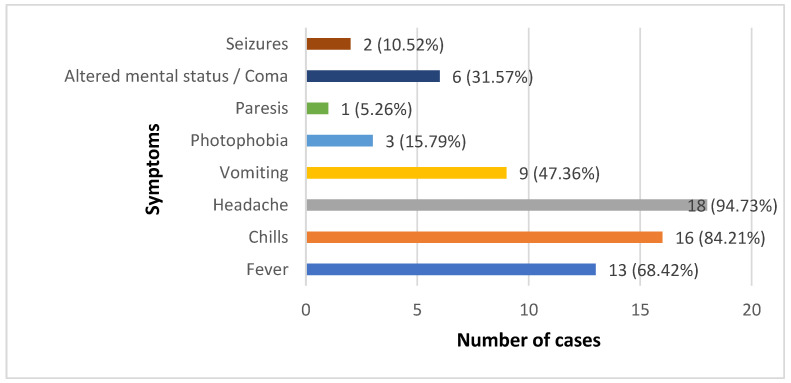
Signs/symptoms at/before hospitalization in our TBM patients.

A majority of patients had fever (13/19) and/or chills (16/19) during this period, but usually their body temperature did not exceed 38 °C (only 2 patients had a higher fever). Headache was the main clinical sign, being present in 18/19 patients. A total of 6 of the 19 TBM patients had an altered mental status at admission (defined as a Glasgow coma scale below 10 points) and, of these, two were in a deep coma (Glasgow coma scale 4 and 6). Vomiting was also a frequent symptom in these patients; photophobia, the presence of seizures or paresis, was less frequently seen (Figure 1).

The most frequent associated infectious pathology registered, other than HIV and TB, was oropharyngeal candidiasis (seven cases, 36.8%), followed by chronic hepatitis B virus (four cases, 21.1%); two chronic hepatitis C virus, and one syphilis coinfection were recorded (Table 3).

**Figure 2:**
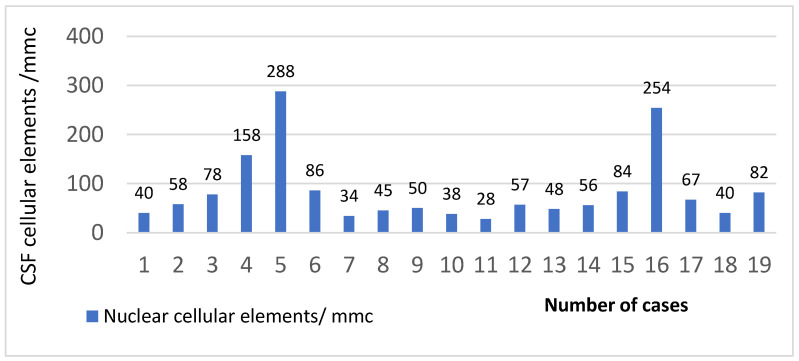
The quantitative examination of cerebrospinal fluid in HIV–TBM study cases.

The quantitative cytological examination of the CSF showed that the majority of the patients had pleocytosis below 100 leukocytes/mm^3^ (16 cases, 84.21%), and only 3 cases had more leukocytes in their CSF at admission (Figure 2). The mean value of the nuclear cellular elements was 83.73 cells/mmc.

**Figure 3:**
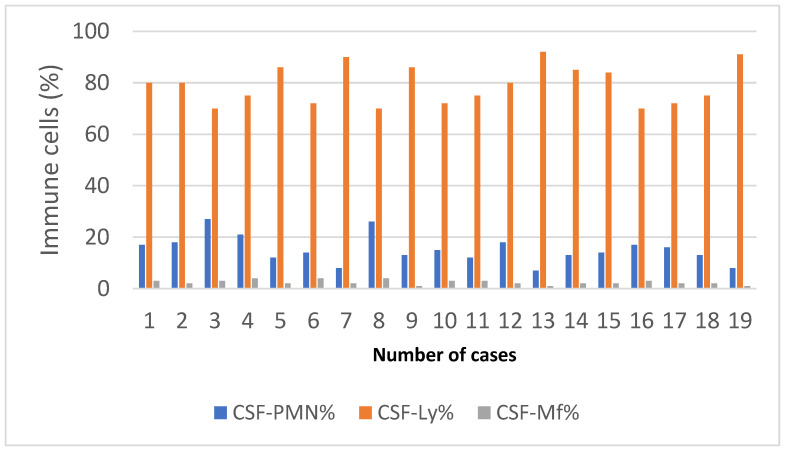
The qualitative examination of cerebrospinal fluid in HIV–TBM study cases.

The qualitative cytological examination showed lymphocytic predominance with decreased levels of neutrophils and macrophages. The lymphocyte range was between 70% and 92%, with a mean value of 79.21%. The neutrophil level was between 7% and 27%, and the mean value was 15.21%. Macrophages were contained between 1% and 4%, and the average value was 2.42% (Figure 3).

**Figure 4:**
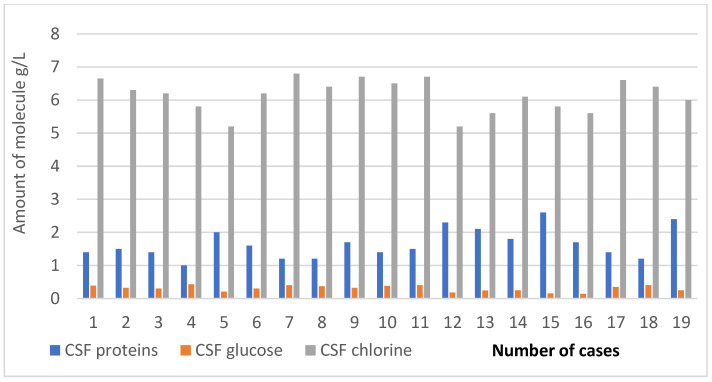
Biochemistry examination of cerebrospinal fluid of HIV–TBM study cases.

The evaluation of CSF biochemistry revealed increased protein levels that ranged between 1.0 g/L and 2.1 g/L, with a mean value of 1.65 g/L. The CSF glucose was decreased, with values between 0.14 g/L and 0.43 g/L; the average value was 0.30 g/L. The CSF chlorine was also decreased, with values between 5.2 g/L and 6.8 g/L, with a mean value of 6.14 g/L (Figure 4). The CSF/plasma glucose ratio was calculated, with values ranging from 0.30 to 0.44, suggesting a bacterial rather than a viral etiology. The mean value for the CSF/plasma glucose ratio was 0.39.

The TBM–HIV patients presented a deficient viro-immunological status. Almost all patients had less than 200 CD4+ T-lymphocytes/mm^3^ (89.47%, 17 cases). Only two cases (10.52%) had a CD4+ T-lymphocyte value between 200 and 499 cells/μL. The median CD4+ T-lymphocyte level of the TBM cases was 47 cells/μL. The average HIV viral load was 403,844.2 copies/mL.

The CD4+ T-lymphocyte level distribution correlated with gender showed that the most affected were females, with a lower CD4+ T-lymphocyte level overall. 

In the study group, it was observed that one-third of the males had a high ALT value (15.78%), and almost half had a high AST (21.05%); as for the females, almost one-third had an increased value of ALT or AST (15.78% and 21.05%, respectively). From this, 15.78% of males had a high ALT/AST value, 5.26% (one case) had hepatitis B, 5.26% (one case) had hepatitis C, and only 5.26% (one case) declared occasional alcohol consumption. Furthermore, in 31.56% of female patients with high AST/ALT values, 15.78% (three cases) had hepatitis B, 5.26% (one case) had hepatitis C, and only 10.52% (two cases) declared occasional alcohol consumption. The rest of the patients did not have an identified cause of elevated transaminase values (Table 4; N, normal value; H, high value).

Thwaites’ system [18] was used to differentiate TBM from other bacterial meningitis cases. The score values calculated advocated for tuberculous meningitis, at values of ≤4. As a result of their deficient viro-immunological status, our patients had low white blood cell counts. Most of the patients had pleocytosis of the order of “tens” (16 cases), and three cases had pleocytosis of the order of “hundreds” (the highest value was 288 cells/mmc). The neutrophil level was between 7% and 27%, with a mean value of 15.21%. In total, 16 patients presented a history of illness for more than 6 days (Table 5). Only two patients had a score above 4 points. Most patients (14) had -5 points, two had a score of -3, and one had a score of 4 points. This resulted in 89.5% sensitivity of the Thwaites scoring system in our cohort.

Prior to the TBM infection, the ART of the HIV-infected patients included protease inhibitors (seven cases, 46.15%); others had integrase inhibitor regimens (five cases, 38.46%), and one case (7.69%) had classic combined therapy (two non-nucleoside reverse transcriptase inhibitors and one nucleoside reverse transcriptase inhibitor) (Table 6).

Anti-tuberculous treatment was established following the European AIDS Clinical Society (EACS) recommendation. The treatment strategy included quadruple association with 2 months of daily isoniazid, rifampin, pyrazinamide, and ethambutol, followed by 7–10 months of isoniazid and rifampin, according to the guidelines. A standard dose of rifampicin (10 mg/kg/day, maximum 600 mg/day) was used.

There were no registered drug-resistant tuberculosis cases, according to the Xpert MTB/RIF PCR assay performed or significant treatment-related adverse effects. 

In the newly diagnosed HIV late-presenting cases, ART was initiated after 2–3 weeks of TBM treatment, depending on the clinical evolution of HIV–TBM cases.

The evolution of the cases was favorable in 13 cases, and 6 patients died. The immune status of the deceased patients was significantly worse than that of the survivors (mean CD4 count 8 vs. 91.2, *p* = 0.034). Increased lethality risk was observed in late presenters with CD4 levels under 200 cells/mmc; four of the six late presenters died, compared to two of the thirteen patients with previously known HIV infection.

The in-hospital lethality rate among the non-HIV TBM patients was 10.7%.

Among the surviving patients, neurological sequelae after TBM were not recorded; they continued to follow the anti-tuberculous treatment according to the indications of the pulmonologist and maintained the antiretroviral treatment.

Most of the patients needed more than 20 days of hospitalization (10 cases (52.63%)); 7 cases (36.84%) were admitted for between 11 and 20 days, and 2 cases (10.52%) were admitted for between 0 and 10 days. The median admission period was 18 days.

The surviving patients were evaluated 3 months after discharge, and the viro-immunological status showed an increasing CD4+ T-lymphocyte level and a significant decrease in HIV viremia. The average HIV viral load was 20.61 copies/mL, and only two patients (15.38%) had detectable HIV viremia, but under 1000 copies/mL. Most cases had a CD4+ T-lymphocyte level > 500 cells/μL (Table 7).

## 4. Discussion

TB remains one of the most significant and socially neglected diseases because of its associations with prejudice, delayed diagnosis, limited access to therapies, and subpar follow-up. When linked to HIV infection, it significantly challenges the global and national health systems [19].

Thwaites et al. [20] showed that extrapulmonary tuberculosis was more likely to develop in HIV-infected patients. In our study, the annual number of TBM–HIV coinfected patients remained relatively constant throughout our study period, although the number of HIV–TB coinfected and non-HIV TB patients significantly decreased in the second half of that period. They were, on average, younger than the non-TBM HIV patients from our center (27 vs. 36 years old, *p* = 0.0007).

Our patients presented an altered viro-immunological status (with a median CD4+ lymphocyte count of 47 cells/mm^3^), a favorable condition for opportunistic infections, particularly TBM. The risk of TBM in an HIV-positive person increases, especially when immunosuppression is further established [21]. A study by Cresswell et al. [22] enrolled 61 TBM adults (92% were living with HIV) with a median CD4 count of 50 cells/μL, and the median number of CD4+ cells/μL in Croda’s study [23] was 65 cells/μL vs. 47 cells/μL in our study. Our HIV–TBM patients have significantly lower levels of CD4 lymphocytes compared with the rest of the HIV cohort under our care. Also, patients with TBM and HIV tend to be younger than those without M. tuberculosis coinfection (Table 3); similar results were reported by Boonyagars [24]. 

In our study, most patients had typical signs and symptoms that are usually linked to TBM. Several authors also concluded that the clinical and neurological manifestations of TBM are not influenced by the HIV status of the patient [20,24,25]. Fever and chills were present in the majority of our patients, but in a study by Croda et al. [23] that included 108 cases, fever, headache, and meningeal symptoms were only present in 15% of patients. Vinnard reported that clinical traits, including an altered level of consciousness, cerebral infarctions, and a positive M. tuberculosis CSF culture, may be more prevalent in HIV-infected people with TBM [21].

In 2002, Thwaites [18] developed a scoring system based on five clinical and laboratory parameters, in order to permit early differentiation of TBM from other types of meningitis, particularly in settings with limited microbiological resources. A score less than four predicts TBM with 86% sensitivity and 79% specificity. In 2020, Sulaiman [26] reported poor performance of the Thwaites scoring system for a cohort of 395 patients with subacute or chronic meningitis. In our small cohort, only 2/19 patients had a score greater than four, which makes the sensitivity of Thwaites’ scoring system 89.5%, despite the fact that the system was not developed for immunocompromised HIV patients.

Anggraini et al. studied the differences in clinical manifestations, cerebrospinal fluid (CSF) findings, and chest X-ray results between HIV-positive and HIV-negative TBM patients. Only the CSF results had statistical differences. The HIV-positive subjects had higher CSF to blood glucose ratios (0.42 vs. 0.18) and fewer leukocyte cells (41 vs. 199), whilst clinical manifestations and chest X-ray results showed no differences [25]. In our study, the CSF/plasma glucose ratio was calculated, with values ranging from 0.30 to 0.44 and a mean value of 0.39. We also recorded CSF pleocytosis of the order of “tens” (16 cases), and three cases had pleocytosis of the order of “hundreds”. Loscalzo et al. suggest that a CSF/plasma glucose ratio < 0.4 is representative of bacterial meningitis, but can also be found in other etiologies such as tuberculous, fungal, or carcinomatous meningitis [27]. In our study, the CSF/plasma glucose ratio was calculated with values ranging from 0.30 to 0.44 and a mean value of 0.39.

Boonyagars et al. [24] carried out a retrospective cohort analysis on 174 adult patients (55.75% HIV-positive) who received treatment for TBM in Thailand. They concluded that HIV infection does not affect TBM radiological signs, CSF profiles (apart from increased CSF protein in HIV-infected patients), or neurological characteristics, as seen also in our study. We recorded increased CSF proteins and decreased glucose and chlorine in our cohort of patients; these changes are typical for TBM, regardless of the HIV status [7]. Compared to TBM patients who were not infected with HIV, the success rate of treatment was lower in HIV-infected TBM patients. HIV infection lowers the likelihood of successful treatment and survival from an outcome standpoint. We advise early diagnosis and treatment to improve the outcome of TBM treatment [24].

Vinnard et al. [21] concluded that despite the beginning of effective anti-tuberculous therapy, the mortality rates of TBM remain high. Several studies reported a significantly lower survival rate in HIV-infected patients [20,24]. In Croda’s study [23], 9% of cases showed primary isoniazid resistance, and 7% had multidrug-resistant strains; the overall mortality rate was 29% while in the hospital, and 41% after 9 months, and the 9-month survival rate was only 22%. In our study, we did not identify rifampicin resistance; the in-hospital lethality was 31.5%, and the 3-month survival rate was 68.5%. The in-hospital lethality rate of our HIV–TBM patients was significantly higher compared to the non-HIV TBM patients: 31.5% vs. 10.7%, *p* = 0.02. 

Due to the emerging antibiotic resistance of M. tuberculosis, the use of higher doses of rifampicin was proposed by several authors [22,28]. In a study by Cresswell et al. [22], the minimal inhibitory concentration of rifampicin was exceeded in most participants, and at day 14, higher serum and CSF levels were still present. They showed reassuring proof that high-dose rifampicin increases CSF and serum exposures in a population mostly made up of HIV-positive individuals, without causing any additional harm. In our study, as no rifampicin resistance was detected, patients received standard-dose rifampicin treatment regimens, and we also did not record adverse effects. Whether high-dose rifampicin will ever be a part of the optimal TBM treatment regimen is not decided yet [22].

Our surviving patients, after 3 months of combined ART and anti-TB treatment, had a good clinical and immunological response: 61.5% had more than 500 CD4+ T-lymphocytes/μL and 11/13 had undetectable HIV viral load.

There are several limitations of our study: the small cohort of TBM patients, and the fact that the reported cases were from the North East region of Romania and only included patients from Iasi Regional HIV/AIDS Center. For future research, the study could be expanded to include all cases reported in our country. A larger cohort of TBM cases among HIV-infected patients may help in elaborating an algorithm for faster detection and management of the disease. There was no HIV-negative TBM control group, and the follow-up of the patients stopped 3 months after diagnosis.

## 5. Conclusions

The annual number of TBM–HIV coinfected patients remained relatively constant throughout our study period, although the HIV–TB coinfected and non-HIV TB patients significantly decreased in the second half of that period. We found that those who developed tuberculous meningitis were severely immunosuppressed; most of them knew about their HIV infection and were receiving ARV treatment, but there were also six patients in whom TBM and HIV were simultaneously diagnosed. Their median age was lower than that of the rest of the HIV patients in our center, and the clinical manifestations were typical for TBM in most cases. Thwaites’ scoring system had good sensitivity (89.5%) for the prediction of the TBM etiological diagnosis, despite the HIV infection. The management of HIV-positive TBM patients used a multidisciplinary approach, with access to anti-TB and antiretroviral therapy. No rifampicin resistance was detected. The lethality rate was 31.5%, and was linked to a worse immunosuppression status.

## Figures and Tables

**Table 1:** Distribution of TBM cases in HIV-infected people in our regional center.

	n	%
HIV-infected people with TB	195	11.5
HIV + TBM-infected people	19	1.1
HIV-infected people without TB	1478	87.4
Total	1692	100

**Table 2:** Distribution by year of the number of cases of TBM patients with and without HIV coinfection and those with non-meningeal TB and HIV coinfection in our hospital.

Year	2010	2011	2012	2013	2014	2015	2016	2017	2018	2019	2020	2021	2022	Total
TB–HIV	26	35	14	28	24	12	18	11	8	5	6	1	7	195
non-HIV TBM	15	20	10	14	10	5	5	2	2	2	4	1	3	93
TBM–HIV	2	1	2	2	1	1	4	1	1	1	1	0	2	19

**Table 3:** Distribution of the study cohort (TBM–HIV number of cases vs. TB–HIV and HIV cases) based on demographics and viral, immunological factors and comorbidities.

	TBM–HIV (Total = 19)	%	TB–HIV (Total = 195)	%	HIV (Total = 1478)	%	*p*
Gender							
M	9	47.4	109	55.9	854	57.8	0.59
F	10	52.6	86	44.1	624	42.2	
Age group (years)							
0–20	1	5.3	9	4.6	109	7.4	<0.001
21–30	10	52.6	27	13.8	271	18.3	
31–40	5	26.3	48	24.6	592	40.0	
41–50	2	10.5	54	27.7	242	16.4	
51–60	1	5.3	45	23.1	175	11.8	
>61	0	0	12	6.2	89	6.0	
CD4 T cell count (/mm^3^)							
<200	17	89.5	179	91.8	385	26.0	<0.001
200–499	2	10.5	16	8.2	499	33.8	
>500	0	0	0	0	594	40.2	
Associated infections							
OFC	7	36.8	75	38.5	401	27.1	0.005
HBV	4	21.1	49	25.1	298	20.2	
HCV	2	10.5	30	15.4	385	26.0	
Syphilis	1	5.3	14	7.2	106	7.2	
None	5	26.3	27	13.8	288	19.5	

M—male, F—female, HCV—chronic hepatitis C virus, HBV—chronic hepatitis B virus, OFC—oropharyngeal candidiasis.

**Table 4:** Distribution of cases based on gender, metabolic syndrome, and liver enzymes.

Laboratory Marker	Value	Male		Female		Total	
		n	%	n	%	n	%
ALT N: 5–40 UI/L	N	6	31.57	7	36.84	13	68.41
	H	3	15.78	3	15.78	6	31.56
AST N: 5–37 UI/L	N	5	26.31	6	31.57	11	57.88
	H	4	21.05	4	21.05	8	42.10
GGT N: 11–50 UI/L	N	3	15.78	6	31.57	9	47.35
	H	6	31.57	4	21.05	10	52.62
Cholesterol N: 122–200 mg/dL	N	7	36.84	5	26.31	12	63.15
	H	2	10.52	5	26.05	6	36.57
HDL-COLN: >60 mg/dL	N	7	36.84	5	26.31	12	63.15
	H	2	10.52	5	26.05	6	36.57
LDL-COL N: <100 mg/dL	N	5	26.31	3	15.78	8	42.09
	H	4	21.05	7	36.84	11	57.89
Triglycerides N: <150 mg/dL	N	5	26.31	3	15.78	8	42.09
	H	4	21.05	7	36.84	11	57.89

**Table 5:** Thwaites system scoring in our study group.

Variable	No. of Cases
Age (years)	
≥36	5
<36	14
White blood cells (103/mmc)	
≥15.000	2
<15.000	18
History of illness (days)	
≥6	16
<6	3
Total white blood cell count from CSF (103/mmc)	
≥900	0
<900	18
Neutrophils ratio from CSF (%)	
≥75	0
<75	19
No. of cases	Score
14	−5
2	−3
1	4
2	6

**Table 6:** ART of the HIV-infected patients from our center prior to TBM infection.

ART Regimens	Number of Patients	%
Protease inhibitors	7	46.15
Integrase inhibitors	5	38.46
2 INNRT + INRT	1	7.69

**Table 7:** Distribution by CD4+ T-lymphocyte level and HIV viral load after 3 months of ART.

**CD4+ Levels** **(cells/μL)**	**Initial**		**After 3 Months of ART**		***p***
	***n***	**%**	***n***	**%**	
0–200	17	89.5	1	7.69	0.053
200–499	2	10.5	4	30.76	
>500	0	0	8	61.53	
**HIV Viral Load (copies/mL)**	**Initial**		**After 3 Months of ART**		
	***n***	**%**	***n***	**%**	
>10^6^	3	15.8	0	0.0	<0.001
10^5^–10^6^	7	36.8	0	0.0	
10^4^–10^5^	6	31.6	0	0.0	
10^3^–10^4^	2	10.5	0	0.0	
<10^3^	1	5.3	2	15.4	
Undetectable	0	0.0	11	84.6	

## Data Availability

All data generated or analyzed during this study are included in this published article.
